# Diffusion tensor imaging differences relate to memory deficits in diffuse traumatic brain injury

**DOI:** 10.1186/1471-2377-11-24

**Published:** 2011-02-23

**Authors:** Eva M Palacios, Davinia Fernandez-Espejo, Carme Junque, Rocio Sanchez-Carrion, Teresa Roig, Jose M Tormos, Nuria Bargallo, Pere Vendrell

**Affiliations:** 1 Department of Psychiatry and Clinical Psychobiology, University of Barcelona, Barcelona, Spain; 2 Institute of Biomedical Research August Pi i Sunyer (IDIBAPS), Barcelona, Spain; 3 Department of Neuropsychology, Institut Universitari de Neurorehabilitació Guttmann, Badalona, Spain; 4 Centre de Diagnòstic per la Imatge Hospital Clínic de Barcelona (CDIC), Hospital Clínic de Barcelona, Spain

## Abstract

**Background:**

Memory is one of the most impaired functions after traumatic brain injury (TBI). We used diffusion tensor imaging (DTI) to determine the structural basis of memory deficit. We correlated fractional anisotropy (FA) of the fasciculi connecting the main cerebral regions that are involved in declarative and working memory functions.

**Methods:**

Fifteen patients with severe and diffuse TBI and sixteen healthy controls matched by age and years of education were scanned. The neuropsychological assessment included: Letter-number sequencing test (LNS), 2-back task, digit span (forwards and backwards) and the Rivermead profilet. DTI was analyzed by a tract-based spatial statics (TBSS) approach.

**Results:**

Whole brain DTI analysis showed a global decrease in FA values that correlated with the 2-back d-prime index, but not with the Rivermead profile. ROI analysis revealed positive correlations between working memory performance assessed by 2-back d-prime and superior longitudinal fasciculi, corpus callosum, arcuate fasciculi and fornix. Declarative memory assessed by the Rivermead profile scores correlated with the fornix and the corpus callosum.

**Conclusions:**

Diffuse TBI is associated with a general decrease of white matter integrity. Nevertheless deficits in specific memory domains are related to different patterns of white matter damage.

## Background

Diffuse axonal injury (DAI) was initially defined as widespread damage to axons throughout the white matter, evoked by intense shear and strain forces resulting from rapid acceleration and deceleration of the brain with or without impact after traumatic brain injury (TBI) [1,2]. More recently, traumatic axonal injury (TAI) has been suggested as a more appropriate term for describing axonal damage because it encompasses not only the primary axonal damage specifically caused by shear/strain injury, but also secondary alterations of white matter such as metabolic, hypoxic and microvascular damage or excitotoxicity [3,4].

Although TAI has been described in neuropathological terms, magnetic resonance imaging (MRI) allows the detection of microhemorrhages and other indirect signs in regions commonly affected by this injury such as the subcortical white matter, the corpus callosum and the dorsolateral quadrant of the rostral brain-stem. It has recently been demonstrated that T2*-weighted MRI at high field strength is a useful tool for the identification of traumatic microbleeds even in the chronic stage of TBI [5]. However, diffusion tensor imaging (DTI) has been suggested as the best technique for the detection of subtle white matter changes [6] given that it can reveal significant abnormalities in white matter in patients with normal findings in conventional MRI [7,8].

DTI is a non-invasive MRI technique that identifies the microscopic physical properties of tissues directly through the observation of translational molecular movement of water [9]. Water diffusion in cerebral white matter tends to be anisotropic, because the highly linear organization of white matter fibers restricts movement in other directions [10,11]. Fractional anisotropy (FA), one of the main DTI-derived indices, provides information of the degree of directionality of water diffusion and on microstructural white matter changes. DTI has been shown to be an efficient technique for determining white-matter integrity in several pathologies [12]. It has also been proposed as the most feasible biomarker of TAI and one of the best indicators of TBI severity [13,14]. Reductions in FA have been detected not only in moderate and severe TBI patients [15-18] but also in cases of mild TBI [19-24]. Moreover, DTI has proved to be an excellent tool for evaluating structural changes after TBI in longitudinal studies [16-18].

The advantages of DTI have resulted in a growing body of scientific evidence regarding the relationship between white matter damage and neuropsychological deficits in TBI. Studies conducted with pediatric samples have identified correlations between FA values and various cognitive functions, including cognitive processing speed and interference, executive functioning, IQ, verbal working memory, reading comprehension and letter naming speed [25-27]. Recently, Wu et al., [28] reported correlations between immediate recall and left cingulum bundles in adolescents after mild TBI. Some studies have related FA measurements with neuropsychological deficits in adults. Nakayama et al. [29] identified a positive correlation between the Mini-Mental State Examination (MMSE) and FA in the splenium of the corpus callosum. Salmond et al. [30] found a significant correlation between diffusivity and the impairment of learning and memory in the posterior cingulate, hippocampal formation and cortical areas. Kraus et al. [31] in a sample including all grade severities, found reduced FA in the ROIs analyzed and obtained a measure of the total regions of reduced FA that negatively correlated with the three cognitive domains evaluated. Furthermore, in a mild TBI sample, Niogi et al. [32] found a significant correlation between attentional control and FA within a ROI in the corona radiata and between memory performance and FA in the ROI placed in the uncinate both in the group of mild TBI patients and the control group. Kumar et al. [18] found correlations between the corpus callosum and neuropsychological tests involving processing speed as well as visuospatial and visuperceptive tasks. Finally, Lipton et al., [33] and Miles et al. [34] found that reductions in FA in dorsolateral prefrontal cortex correlated significantly with tests of executive functions. In summary, DTI technique, in special FA measures, has been found sensitive to reflect cognitive deficits associate with TBI.

Memory is one of the functions that is most frequently impaired by TBI [35-37]. The concept of multiple memory systems and their different neuroanatomical substrates is currently accepted [38,39]. Declarative and working memory systems are significantly impaired after traumatic brain injury (TBI). Deficits in declarative memory - the capacity for conscious recollection of facts and events - are a common consequence of head trauma that are disproportionately suffered in comparison with other cognitive functions [35,40]. These memory difficulties improve slowly and although progress is made over the first and second year following injury, they remain apparent over time [40-42]. Neuroanatomically, declarative memory depends on the integrity of the hippocampus and its connections with the neocortex [43,44]. In neuroimaging studies with TBI patients, declarative memory has been found to correlate negatively with hippocampal [45,46] and fornix damage [47]. Working memory is defined as the ability to maintain and manipulate information temporarily [48]. Impairment of this memory is frequent in TBI patients given that implicated neural substrates, particularly the frontal cortex, are highly vulnerable in this type of injury. There is considerable evidence that working memory depends on network activity including the frontal and parietal regions and its connections. A meta-analysis of functional neuroimaging studies conducted by Owen et al. [49] provided strong evidence for the activation of frontal and parietal cortical regions by various versions of the n-back paradigm. The main fasciculus linking the parietal and frontal lobes is the superior longitudinal fasciculus (SLF) and hence it is likely that this has a role in working memory. Relations between the SLF and working memory deficits have been reported in multiple sclerosis [50] but not in TBI patients. To our knowledge there is no study investigating the impairment of white matter damage related to declarative and working memory deficits in a sample of severe and diffuse TBI.

The aim of this study was to investigate the role of white matter damage in declarative and working memory deficits after diffuse TBI, focusing on the main associative fasciculi [51] including those connecting the cerebral regions involved in the declarative memory and working memory networks.

Our study had two main hypotheses: firstly, that a decreased FA in the superior longitudinal fasciculi (SLF), which is presumably involved in working memory function since it links the parietal and prefrontal regions, would correlate with working memory deficits, and, secondly, that a decreased FA in the fornix, the main fasciculus interconnecting the hippocampus with the frontal lobe, would correlate with declarative memory impairment.

## Methods

### Subjects

A cross-sectional study of thirty-one subjects was performed. Fifteen patients (eleven male) with severe TBI were recruited from the Head Injury Unit of the Institut de Neurorehabilitació Guttmann. Inclusion criteria were: a) age < 40 years, b) diffuse axonal injury according to clinical MRI without focal cortical lesions or larger than 1.5 cm3, c) severe TBI: defined as a minimal Glasgow Coma Scale (GCS) score ≤ 8 assessed at the first contact with the emergency services, d) emergence from posttraumatic amnesia (PTA) phase at the moment of the enrollment according to the Galveston Orientation and Attention Test (GOAT) [52], defined as two consecutive scores > 65, and f) no previous history of TBI, drug intake, neurological, or psychiatric disorders.

The etiology of TBI was a traffic accident in all cases. Fourteen patients were involved in car collisions, and one was a pedestrian hit by a motor vehicle. All patients had closed head injury and had not received surgery for extra- or subdural hematoma; all structural MRI scans were suggestive of TAI. The neuroradiologist (NB) took into account T1-weighted, FLAIR, and T2* GE sequences. The T2* GE sequences, which have a high level of sensitivity for detecting chronic hemosiderin, indicated evidence of TAI-related neuropathology. The method proposed by Gennarelli et al. [2] was used to classify the patients' TAI type. The grading system used was: type I, TAI only involving convexity gray-white matter junction; type II, also involving the corpus callosum in addition to the gray-white junction; and, type III, involving the rostral brainstem as well as the two previous criteria. Cases in which the midbrain was involved, but no corpus callosum lesions were apparent, were classified as type III (see Table 1).

**Table 1:** Clinical and neuroimaging characteristics of the TBI group

PT	GCS	PTA	Initial CT	MRI findings (T2*/FLAIR-Hemosiderin deposits)	tevol	TAI
**1**	8	150	SAH. Small hemorrhage in L thalamus	Microbleeds in L thalamus, R caudate, midbrain, frontal lobe, and CC	207	III
**2**	7	60	SAH. Small hemorrhagic lesion at the uncus	Microbleeds in R caudate, thalamus, pons, frontoparietal lobes and CC	285	III
**3**	3	125	Small frontobasal contusion and bilateral hemorrhagic foci in R frontal lobe and R thalamus	Microbleeds in R thalamus, R fronto-temporo parietal lobes, hippocampus and CC. Frontobasal contusion (< 1.5 cm)	315	II
**4**	5	45	No evidence of lesions	Microbleeds in dorsal midbrain and L frontal lobe	429	III
**5**	4	40	SAH. Hemorrhagic focus in L frontal white matter	Microbleeds in midbrain, R/L hippocampus, frontal and temporal lobes, and CC	550	III
**6**	7	51	SAH. Hemorrhagic focus in the R frontal white matter, and R intraventricular hemorrhage	Microbleeds in R/L hippocampus and R prefrontal region	146	I
**7**	4	45	Hyperdense lesion in the L medial temporal lobe	Microbleeds in L caudate, R/L hippocampus, midbrain, L parietal, R frontal lobes and CC	165	III
**8**	4	75	Multiple small bilateral subcortical hemorrhagic foci	Microbleeds in L thalamus, R midbrain, cerebellar peduncle, R/L frontal parietal and occipital lobes, R temporal and CC. Fonto-temporal deep white matter hyperintensities due to demyelination	86	III
**9**	3	70	Small hemorrhagic foci at R internal capsular and temporal region, and L CC. Intraventricular hemorrhage	Microbleeds in midbrain, cerebellum, R hippocampus, R internal capsule and thalamus, L fronto-parietal, and CC	443	III
**10**	3	60	SAH. Diffuse white matter alterations	Microbleeds in R thalamus, R midbrain, cerebellum, R/L frontal and CC. Parietal deep white matter hyperintensities due to demyelination.	114	III
**11**	7	120	Multiple puntiform hemorrhagic foci in both hemispheres	Microbleeds in L thalamus, R globus pallidus, R/L insula, R midbrain R/L frontal, parietal and temporal lobes and CC	306	III
**12**	4	171	Puntiform temporal contusion. L temporal subdural hematoma	Multiple subcortical microbleeds in pyramidal tract, centrum semiovale, pons, and CC. Deep white matter lesions. L temporal contusion (< 1 cm)	213	III
**13**	8	20	No evidence of lesions	Microbleeds in L insula, R frontal lobe and CC. Deep white matter lesions predominantly in the parietal lobe	115	II
**14**	4	105	Multiple hemorrhagic foci	Microbleeds in midbrain, fronto-parieto-occipital lobes and CC. Deep frontal white matter hyperintensities due to demyelination	143	III
**15**	6	120	Microhemorrhages in the L cerebellar hemisphere and R frontal lobe	Microbleeds in R thalamus, R temporal lobe and in fronto-parietal lobes. Contusion in R frontal gyrus and frontobasal (< 1.2 cm)	660	II

PT: Patient; GCS: Glasgow coma scale; PTA: posttraumatic amnesia; CT: computer tomography; MRI: magnetic resonance imaging; tevol: time of evolution since accident to the MRI evaluation; TAI: diffuse axonal injury; R/L: right/left; CC: corpus callosum; SHA: subarachnoidal hemorrhage.

A control group of sixteen healthy subjects (nine male) were recruited from relatives and friends of the TBI group. This control group was matched by age, years of education and premorbid intellectual function estimated using the Vocabulary subtest of the Wechsler Adult Intelligence Scale (WAIS-III) [53], recognized as an efficient method for estimating general intelligence [54] (see Table 2). All subjects were right-handed, Caucasian-Mediterranean, and none had a previous history of neurological or psychiatric diseases.

**Table 2:** Demographic and clinical characteristics of TBI and control groups

	TBI group	(n = 15)	Control group	(n = 16)
	Mean	SD (Range)	Mean	SD (Range)
**Age**	23.6	4.79 (18-32)	23.7	4.8 (18-32)
**Education (years)**	11.3	2.7 (8-16)	11.9	2.8 (8-16)
**Vocabulary (WAIS-III)**	9.9	2.0 (8-14)	10.3	1.9 (8-14)

TBI = traumatic brain injury.

The study was approved by the Ethical and Research Committee of the Institut Universitari de Neurorehabilitacio Guttmann and all participants gave written informed consent.

### Memory assessment

Working memory was evaluated by the Digit span and Letter-Number Sequencing (LNS) subtests of the WAIS-III [53] and a visual 2-back task [55]. Digit span was measured as the series length correctly reproduced at least once in the same order (forwards) and in reverse order (backwards). In the LNS, subjects heard lists of randomized numbers and letters (in alternating order) of increasing lengths, and were asked to reproduce the numbers and letters beginning with the lowest in each series, always with numbers first. The scores from the LNS were calculated by adding all correct items. In the 2-back task, numbers appeared on the screen for 500 ms against a black background, followed by a fixation cross for 1500 ms. The subjects were asked to decide whether the number they were looking at matched the one that they had seen two numbers earlier in the sequence. The numbers of correct responses as well as the reaction time were recorded. The d-prime index, a bias-free measure that takes both correct answers and errors into account, was also calculated to determine the accuracy of performance.

The Rivermead Behavioural Memory Test (RBMT) [56] was selected for its ability to explore declarative memory, and its ecological validity in assessing TBI patients. This test consists of 11 subtests including the following: remembering a name, a hidden belonging and an appointment; recognizing pictures and faces; recalling a prose passage; remembering a short route; remembering to deliver a message; and knowledge of some basic information such as the date, place and time. Those are designed as analogs of everyday tasks, reflecting the kinds of situations with which patients typically experience difficulty on a day-to-day basis. Two methods of standardizing scores across subtests allow for derivation of either a screening score, with subtest raw scores categorized on a scale of 0 ± 1 (maximum score 12 points), or a standardized profile score, with subtest raw scores categorized on a scale of 0 ± 2 (maximum score 24 points). The slightly more fine-grained standardized profile score provides a more sensitive analysis of performance [57] thus we use this score for our correlation analysis.

### Image acquisition and analysis

MRI data sets were acquired on a 1.5 T Signa GE (*General Electric, Milwaukee, WI*) at the Centre de Diagnostic per la Imatge of the Hospital Clínic (CDIC), Barcelona. Diffusion weighted images were sensitized in 25 non-collinear directions with a b-value = 1000 sec/mm^2^, using an echo-planar (EPI) sequence (TR = 9999.996 ms, TE = 85 ms, 20 axial slices with a resolution of 0.9375 × 0.9375 mm, slice thickness = 5 mm, gap = 2 mm matrix size = 128 × 128, FOV = 100).

Data preprocessing and analysis was performed using FMRIB's software library [FSL version 4.1; Oxford Centre for Functional MRI of the Brain (FMRIB), UK; http://www.fmrib.ox.ac.uk/fsl/]. Image artefacts due to eddy current distortions were minimized by registering the diffusion images to the b0 images. The registered images were skull-stripped using the Brain Extraction Tool (BET) [58]. Fractional anisotropy maps were calculated using the FMRIB's Diffusion Toolbox v.2.0 [FDT, [59]]. After calculation of the FA map for each subject, we implemented a voxel-wise statistical analysis of the FA data using Tract-Based Spatial Statistics v1.2 (TBSS) which aims to overcome the limitations of the standard VBM-style analyses [60], particularly those regarding to its dependence on the goodness of the registration algorithm and on the choice of the spatial smoothing [61]. FA data were aligned into a common space using a non-linear registration algorithm (FNIRT) to register the images to the standard FMRIB58 FA template, which is in MNI152 standard space. Aligned FA maps were visually inspected after registration and we confirmed that the result of the previous step was correct. Next, a mean FA image was created from the images from all the subjects in this common space and narrowed to generate a mean FA skeleton that represented the center of all tracts common to the entire group. This was thresholded to FA 0.2 to include the major white matter pathways but to exclude peripheral tracts where there was significant inter-subject variability and partial volume effects with gray matter. This ensured that each subject's skeleton was in the group space while also representing the center of the subject's unique white matter bundles. The aligned FA image for each subject was then projected onto the skeleton by filling the skeleton with FA values from the nearest relevant tract centre. This is achieved for each skeleton voxel by searching perpendicular to the local skeleton structure for the maximum value in the FA image of the subject. The resulting skeletonised data was then fed into voxelwise cross-subject statistics.

### Statistical analysis

Group comparisons and correlations with neuropsychological measures were performed using Randomise v2.1 from FSL [62,63]. As seen in table one, the time of evolution since injury was very heterogeneous. In order to control possible effects of this variable in the correlation results, time of evolution was entered as a non-interest variable in the matrix. The statistical threshold was set at p < 0.05 Family Wise Error (FWE) corrected, which is a conservative procedure that allows a high control of Type I error, being the probability of one or more false positives the same as the significance level. The Threshold-Free Cluster Enhancement (TFCE) method was used to define the clusters [64]. Correlation analyses were performed with the 2-back d-prime index and the Rivermead profile score using a region of interest (ROI) approach in the following associative fasciculi: corpus callosum, superior and inferior longitudinal, inferior fronto-occipital, uncinate, and cingulate as well as the fornix and arcuate fasciculi as the major pathways that connect associative cortical regions involved in working and declarative memory. ROI masks were obtained from the Jülich histological atlas [65,66] and the JHU white-matter tractography atlas [67-69]. Areas corresponding to significant clusters were identified using the JHU white-matter tractography atlas. Mean FA values were obtained from each subject's FA skeleton map and skeletonised SLF and fornix ROIs. Mean FA values were obtained from each subject's FA skeleton map and skeletonized for all the fasciculi ROIs mentioned above.

Statistical tests on non-imaging data were performed using SPSS (*Statistical Package for the Social Sciences) v.16 *(SPSS Inc., Chicago Illinois). Group differences were examined using the Student t-test, since the data were normally distributed using a significance level of p < 0.05.

Partial correlation coefficients, controlling for the time of evolution, were used to explore the association between mean FA values and clinical variables and neuropsychological measures. Statistical significance was set at a two-tailed p ≤ 0.05.

## Results

### Comparison between TBI patients and controls

Performance on the memory tests is described in Table 3. Statistical significance was obtained for the difference in scores in the LNS subtest (WAIS-III), d-prime index for the 2-back, and the RBMT profile. Forward and backward digits did not reach statistical significance.

**Table 3:** Neuropsychological performance for TBI and control groups

	TBI	group	Control	group	t (p values)
	Mean	SD	Mean	SD	
**Digit forwards**	6.1	1.0	6.6	1.1	-1.1 (ns)
**Digit backwards**	4.3	1.1	4.9	0.9	-1.6 (ns)
**LNS**	8.6	2.7	11.0	2.8	-2.4 (0.02)
**2- back (d-prime)**	2.7	1.0	3.4	0.4	-2.6 (0.01)
**Goals 2-back**	81.4	18.3	95.2	4.8	-2.7 (0.01)
**Reaction time 2-back (ms)**	693.4	200.0	475.7	88.9	3.7 (0.002)
**RBMT (profile)**	18.1	3.4	22.6	1.3	-4.85 (0.001)

TBI: traumatic brain injury; Letter-Number Sequencing (WAIS-III); Goals: number of targets correctly identified; RBMT: Rivermead Behavioral Memory Test; ns: not significant.

**Figure 1:**
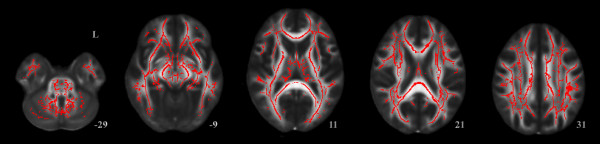
**Results from TBSS analysis of FA maps showing the clusters of significantly reduced FA in TBI patients compared to controls in red (TFCE, p < 0.05 FWE-corrected)**. Widespread white matter affectation is observed.

Group comparison for FA skeleton maps revealed multiple areas of significant FA reductions in TBI patients as compared to controls. All the long associative fibers were affected, including the corpus callosum, the superior and inferior longitudinal fasciculi, and the inferior fronto-occipital fasciculi. Decreased FA was also observed in shorter fibers such as the uncinate fasciculus, cingulum, fornix and anterior thalamic radiation (Figure 1, Table 4). FA was not increased in the TBI group in any cerebral region.

**Table 4:** Differences between groups in mean FA from the whole skeletonised brain and the ROIs

	TBI	group	Control	group	t (p values)
	Mean	SD	Mean	SD	
**FA global**	0.360	0.280	0.423	0.018	-7.43 (< 0.001)
**FA CC**	0.410	0.460	0.510	0.024	-7.04 (< 0.001)
**FA SLF**	0.364	0.026	0.421	0.018	-7.02 (< 0.001)
**FA ILF**	0.045	0.003	0.052	0.002	-7.23 (< 0.001)
**FA IFO**	0.390	0.031	0.456	0.018	-7.45 (< 0.001)
**FA fornix**	0.316	0.030	0.396	0.022	-8.05 (< 0.001)
**FA cingulum**	0.401	0.048	0.493	0.032	-6.16 (< 0.001)
**FA arcuate**	0.373	0.027	0.430	0.018	-6.05 (< 0.001)
**FA uncinate**	0.351	0.032	0.403	0.019	-5.34 (< 0.001)

TBI: Traumatic Brain Injury; FA: fractional anisotropy; CC: corpus callosum; SLF: superior longitudinal fasciculi; ILF: inferior longitudinal fasciculi; IFO: inferior fronto-occipital fasciculi.

We obtained mean FA values of the whole skeletonized brain and all of the selected ROIs. Group comparisons for all these values reached statistical significance in all cases with p < 0.001 (Table 4).

### Correlation analysis

#### Correlation with clinical variables

We observed significant negative correlations between FA and posttraumatic amnesia (PTA) in almost all the regions that showed significant FA decreases in the group analysis. Quantitative global mean FA values also showed a high correlation with this variable (r = -0.903 p <0.001). However, no significant correlations were found in the FA maps analysis for the GCS (*r*= 0.206, p = 0.499).

#### Correlation with declarative and working memory performance

The mean global FA measure correlated significantly with 2-back d-prime index (*r *= 0.584, p = 0.028). The correlation of global FA with the RBMT profile score did not reach statistical significance.

**Figure 2:**
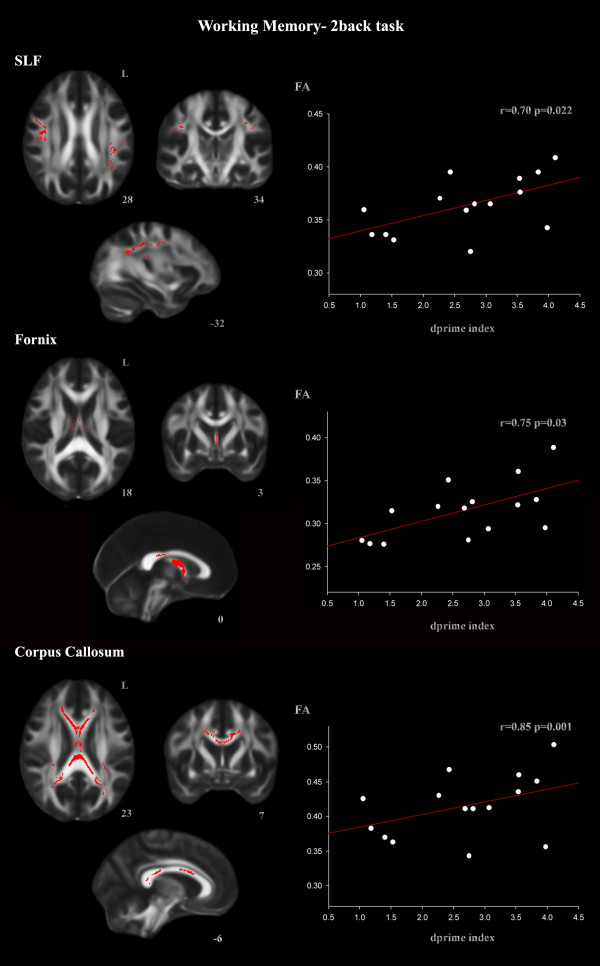
**ROI correlations with d-prime 2-back index in the TBI group for the SLF, fornix, and corpus callosum ROIs (TFCE, p < 0.05 FWE-corrected)**. Correlation coefficient (*r) *was directly converted from t values of the TBSS output. The *t *and *r *values correspond to the most statistically significant voxel for each cluster.

**Figure 3:**
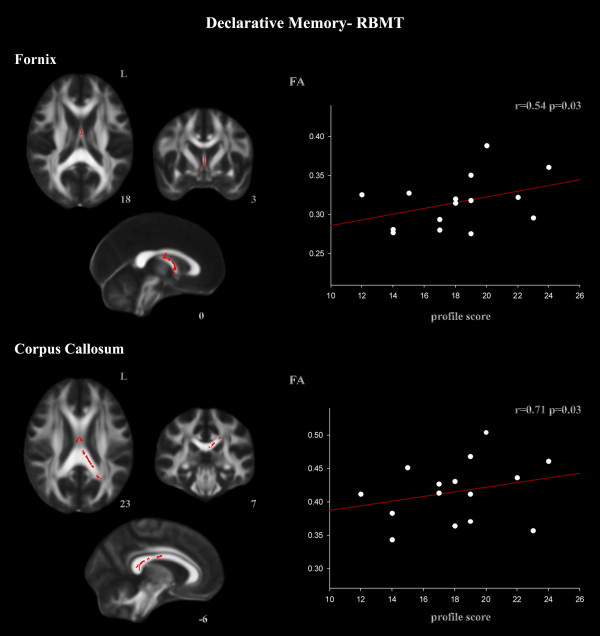
**ROI correlations with RBMT in the TBI group: RBMT correlated with the fornix and the corpus callosum ROIs (TFCE, p < 0.05 FWE-corrected)**. Correlation coefficient *(r) *was directly converted from *t *values of the TBSS output. The *t *and *r *values correspond to the most statistically significant voxel for each cluster.

The ROI procedure revealed a positive correlation between working memory performance assessed by the 2-back d- index and the FA skeletonized SLF, fornix, and corpus callosum ROIs (Figure 2, Table 5). 2-back d-prime index also correlated with the arcuate fascicle (Table 5). Declarative memory performance, assessed by RBMT, correlated with the fornix and the posterior part of the corpus callosum ROIs (Figure 3, Table 5).

**Table 5:** TBSS results. Correlation with working and declarative memory measures in the ROIs in the TBI group

2-back d-prime index	Cluster size	*x*	*y*	*z*	p	r
Superior longitudinal fasciculus	908	-38	-49	22	0.022	0.70
	440	51	2	24	0.025	0.71
	91	-29	-7	39	0.044	0.67
	27	-31	-14	40	0.045	0.69
	20	-46	-46	23	0.048	0.69
Arcuate fasciculus	121	50	3	27	0.030	0.75
	45	-34	-32	35	0.044	0.64
	30	-38	-49	23	0.045	0.73
	16	-36	-29	27	0.048	0.63
Corpus callosum	3310	-7	14	22	0.028	0.65
	2195	-13	-39	23	0.021	0.77
	211	-32	-41	16	0.043	0.67
Fornix	1206	-9	-14	13	0.001	0.85
						
**RBMT**	**Cluster size**	***x***	***y***	***z***	**p**	**r**
						
Corpus callosum	1008	-16	-40	27	0.033	0,71
	538	7	-12	25	0.042	0.68
Fornix	140	-2	-12	-24	0.034	0,54
	68	1	5	-4	0.042	0.60

All regions identified at family wise error corrected p < 0.05 with a cluster size of at least 10 voxels. Coordinates are given at peak voxel coordinate in MNI-152 standard space.

No other correlation reached statistical significance in the TBI group. There were no significant correlations between FA and neuropsychological measures in the control group.

## Discussion

The present study provides evidence of the implications of TAI in declarative and working memory deficits in TBI. DTI group comparison revealed global whole brain reductions in mean FA values for patients and FA maps confirmed that almost all the major fibers were involved. Although our patients suffered global white matter integrity impairment, we found two different and restricted patterns of correlations with the FA and neuropsychological assessment. Whole brain DTI analysis showed that decreased FA throughout the brain correlated with 2-back measures but not with the Rivermead Test. Results from the ROI analyses of the main association fibers showed, as predicted, that working memory specifically correlated with the superior longitudinal fasciculi. However, it was also found to correlate with the corpus callosum, the arcuate fasciculi and with the fornix. On the other hand, declarative memory deficits only correlated with the fornix, as we had expected, and the corpus callosum. These results suggest that there are two different patterns of FA reduction related with two types of memory dysfunctions.

We found that superior longitudinal fasciculi damage is related with working memory but not with declarative memory deficits. These correlations were expectable since the longitudinal fasciculi connect the associative frontal and parietal regions involved in working memory functions [70-72,49]. The correlation between working memory deficit and the superior longitudinal was also described in multiple sclerosis, pathology that also involves white matter damage [50].

In our sample, FA reductions of corpus callosum correlated with both working and declarative memory impairments. In declarative memory the correlations were seen in the posterior region whereas in working memory the correlations involved anterior and posterior regions, thus again these results point to differential patterns of correlations for both types of memory impairment.

According our results, declarative memory impairment did not depend on diffuse white matter damage since no correlations between FA maps or mean values and declarative memory values were seen. However, the ROI analysis revealed that the fornix FA impairment correlated with the Rivermead test. This result is in agreement with the role of the damage of the hippocampus and its connections in declarative memory deficits in TBI [45,46].

Our declarative memory results partially agree with those obtained by Salmond et al. [30]. Using a voxel-based analysis with SPM tools, these authors found a significant positive correlation between declarative memory and diffusivity in the left hippocampal formation, the left posterior cingulate, and the left frontal, temporal and occipital regions. The more widespread pattern of correlations observed in their study can be explained by the use of FDR correction, which is more liberal than the FWE correction used in ours [73]. Correlations between FA values in the fornix and declarative memory impairment have been also observed in patients with multiple sclerosis [74]. Other studies investigating FA correlations with declarative memory functions in mild TBI samples have reported significant correlations with the uncinate fasciculi [30,32] and the cingulum [28]. Although we found decreased FA in these fasciculi, correlations did not reach statistical significance. These discrepancies may be explained by the varying grade of severity of the samples, the difference in the memory tests used, and DTI methodological differences.

In the present study, working memory deficits also correlated with the fornix in both the whole brain analysis and the ROI analyses. There is some evidence from fMRI studies that the hippocampus is involved in working memory functions in healthy subjects [75-78]. Moreover, several animal studies also suggest a role for the hippocampus in working memory [79-81]. Anatomically, prefrontal regions involved in working memory tasks receive projections from the hippocampus [82,83] and are connected directly to the ventral hippocampus and indirectly to the dorsal hippocampus via the thalamus [84-86]. This structural connectivity supports the idea that the hippocampus has a role in working memory functioning as suggested by our findings.

Finally, significant correlations were observed between the PTA variable and white matter integrity. Whole brain map analysis showed that PTA is an excellent index predictor of the degree of impairment of the major white matter tracts and association fibers. These results suggest that the recovery of memory functions is dependent on the integrity of the complex neocortical regions. Unlike previous studies [13,14,17], no correlations were found between GCS and FA maps or mean FA values. This result was to be expected as the fact that all our patients had severe TBI meant that GCS variability would not be sufficient to reach statistical significance.

Our study has certain limitations and our results should be regarded as preliminary. The small sample size and its specific diffuse characteristics may preclude the generalization of the results. The presence of mixed focal and diffuse pathology frequently observed in severe TBI may confound the mapping of neural and behavioral changes in these patients. As our study sample excluded significant cortical pathology, the cognitive impairment observed is more likely to be due to the diffuse pathology alone. Nevertheless, we cannot exclude the possibility that reductions in gray matter in several subcortical structures are also influencing memory deficits in TBI.

## Conclusions

This DTI study suggests that declarative and working memory deficits in diffuse TBI patients are related to differential patterns of FA reduction. Working memory impairment reflects the diffuse white matter damage affecting large scale networks such as the superior longitudinal fasciculi, whereas declarative memory deficits seem to be the result of more local disruption of the cerebral circuitry.

## Competing interests

The authors declare that they have no competing interests.

## Authors' contributions

EP, DFE and CJ made substantial contribution to conception and design, interpretation of data, drafting and writing of manuscript and further revisions of the manuscript. Neuroimaging data were analyzed by EP and DFE. Neuroimaging sequence acquisitions, neurological description and classification of data by NB. RSC and TR participated in the collection of neuropsychological data and in the collection of acute clinical data. JT and PV made a critical revision of the manuscript for important intellectual content providing additional comments and contributions. CJ supervised the study. All authors read and approved the final manuscript.

## Pre-publication history

The pre-publication history for this paper can be accessed here:

http://www.biomedcentral.com/1471-2377/11/24/prepub
